# Towards Reliability in Smart Water Sensing Technology: Evaluating Classical Machine Learning Models for Outlier Detection

**DOI:** 10.3390/s24134084

**Published:** 2024-06-24

**Authors:** Mimoun Lamrini, Bilal Ben Mahria, Mohamed Yassin Chkouri, Abdellah Touhafi

**Affiliations:** 1 Department of Engineering Sciences and Technology (INDI), Vrije Universiteit Brussel (VUB), 1050 Brussels, Belgium; abdellah.touhafi@vub.be; 2 SIGL Laboratory, National School of Applied Sciences of Tetuan, Abdelmalek Essaadi University, Tetuan 93000, Morocco; mychkouri@uae.ac.ma; 3 SIA Laboratory, Faculty of Science and Technology, Sidi Mohamed Ben Abdellah University, Fez 30000, Morocco; 4 Department of Electronics and Informatics (ETRO), Vrije Universiteit Brussel (VUB), 1050 Brussels, Belgium

**Keywords:** anomaly detection, machine learning, sensors of water technology

## Abstract

In recent years, smart water sensing technology has played a crucial role in water management, addressing the pressing need for efficient monitoring and control of water resources analysis. The challenge in smart water sensing technology resides in ensuring the reliability and accuracy of the data collected by sensors. Outliers are a well-known problem in smart sensing as they can negatively affect the viability of useful analysis and make it difficult to evaluate pertinent data. In this study, we evaluate the performance of four sensors: electrical conductivity (EC), dissolved oxygen (DO), temperature (Temp), and pH. We implement four classical machine learning models: support vector machine (SVM), artifical neural network (ANN), decision tree (DT), and isolated forest (iForest)-based outlier detection as a pre-processing step before visualizing the data. The dataset was collected by a real-time smart water sensing monitoring system installed in Brussels’ lakes, rivers, and ponds. The obtained results clearly show that the SVM outperforms the other models, showing 98.38% $F_{1}$-score rates for pH, 96.98% $F_{1}$-score rates for temp, 97.88% $F_{1}$-score rates for DO, and 98.11% $F_{1}$-score rates for EC. Furthermore, ANN also achieves a significant results, establishing it as a viable alternative.

## 1. Introduction

In recent years, the advancement of smart water sensing technology has revolutionized water management practices, offering efficient solutions for monitoring and controlling water resources. Poor water quality poses a severe threat to human health, ecosystems, plants, and animals, emphasizing the critical importance of monitoring water quality in environmental systems [1]. Traditionally, manual laboratory-based testing has been the primary method for water quality monitoring, but it is often costly, time-consuming, and lacks real-time feedback. Thus, the intersection of technology and environmental sustainability has become imperative for ensuring efficient water management [2].

Smart water sensing technology represents a pivotal milestone in water management, leveraging data collected by various sensors deployed in diverse aquatic environments. However, the reliability and accuracy of the collected data are often confounded by anomalies caused by technical issues, which can hinder fault detection and diagnosis [1,3]. Addressing this challenge, our study focuses on detecting outliers to ensure efficient water management, employing machine learning techniques for anomaly detection [4].

Specifically, we evaluate the performance of four key sensors pH, Dissolved Oxygen (DO), Electrical Conductivity (EC), and Temperature (Temp) using classical Machine Learning (ML) algorithms: Support Vector Machine (SVM), Artificial Neural Network (ANN), Decision Tree (DT), and Isolated Forest-based (iForest) outlier detection. Our evaluation is based on data collected from a real-time smart water sensing monitoring system deployed across various environments in Brussels, including lakes, rivers, and ponds.

Our experimental findings underscore the superior efficiency of SVM, demonstrating exceptional $F_{1}$-score rates across multiple sensors. Additionally, we highlight the noteworthy performance of ANN, suggesting its potential capability as a primary alternative for addressing the challenge of outlier detection in water quality monitoring.

In this paper, we present a comprehensive exploration of outlier detection techniques in the context of smart water sensing technology. By assessing the efficacy of classical ML models, we aim to provide valuable insights into enhancing the reliability and accuracy of water quality data analysis, thus contributing to the advancement of water resource management practices.

The rest of this paper is organized as follows: The related study that is relevant to our issue is covered in Section 2. Section 3 an overview of the various types of anomalies is provided, and different indicators have been used to classify anomaly detection techniques. In Section 4 we provide a description of the sensors used in this research as well as the water quality standards provided by the world health organization. The evaluation methodology is also provided in Section 5. In Section 6 outline the evaluation flow of the experiments and the experimental setup utilized. Section 7 contains the results and discussion of the experimental. Finally, we conclude this paper in Section 8.

## 2. Related Work

We conducted a detailed analysis of the literature on ML-based approaches and anomaly detection used in water sensor technology, this domain has gained more attention in recent years due to the increasing importance of this vital substance. Furthermore, this section covers the critical aspects of collection data and pre-processing steps. It aims to provide an overview of the literature concerning the implementation of sensors, communication and research on the ML-techniques for smart water technologies. Our main objectives were to understand about the most effective approaches being used and to identify existing gaps.

Anomalies within water quality data from river sensors can be automatically identified using advanced detection methods. The authors in [5] demonstrate that distinct anomalies, such as abrupt spikes and level alterations, can be reliably detected using regression-based techniques. However, these techniques become less effective when multiple water quality parameters are analyzed concurrently. Their framework focuses on automated anomaly detection within high-frequency datasets, particularly for turbidity, conductivity, and river level metrics from rivers feeding into the Great Barrier Reef. Similarly, the study by [6] addresses the critical need to mitigate the spread of harmful substances within river systems. The authors propose a methodology that combines a Bayesian autoregressive model with the Isolation Forest algorithm, effectively predicting variations in water quality and detecting anomalies. In another notable study, ref. [7] evaluates different outlier detection methods for water sensor data from Dutch water authorities. Among these methods, the QR-MLP model is identified as the most effective, detecting both simulated and real anomalies without requiring specific rules for each type of outlier. ML techniques have proven to be powerful tools in the detection of anomalies in water quality data. The study in [8] investigates ML techniques for detecting anomalies in water quality data from a treatment plant in Kenya. It highlights the Local Outlier Factor algorithm as particularly effective for identifying outliers in turbidity and pH parameters and compares four time-series anomaly detection methods. Further expanding on anomaly detection in water treatment, ref. [4] compares Deep Neural Networks (DNNs) and Support Vector Machines (SVMs) within a Cyber-Physical System (CPS). The study finds that while DNNs produce fewer false positives, SVMs detect slightly more anomalies, indicating that each method has distinct advantages depending on the application context. The application of the One-Class Support Vector Machine (OC-SVM) technique in water supply data is explored in [9], where high abnormality scores are attributed to entries with extreme values and unusual sequences. This study, based on data from the Ministry of Water Resources of China, highlights the effectiveness of OC-SVM in identifying significant anomalies. Real-time monitoring for drinking water quality is crucial, as illustrated in [10], which proposes the ADWICE data mining algorithm. Integrated into SCADA systems, this algorithm enhances anomaly detection accuracy by analyzing sensor data from multiple locations, addressing issues such as missing and inaccurate data. Building on the importance of early and accurate anomaly detection, in [11], the OddWater method utilizes statistical transformations and unsupervised outlier scoring to identify outliers caused by technical errors in sensor data, maintaining low false detection rates. This method is particularly effective for early detection in water quality monitoring systems. An innovative approach in environmental monitoring is presented in [12], where machine learning techniques and correlated sensor data improve outlier detection. By modeling individual sensor behavior and leveraging data correlations, this method significantly enhances data quality and reliability. For smart city applications, ref. [13] emphasizes the use of low-cost, low-power IoT sensors for water monitoring. The proposed anomaly detection system employs one-class classifiers to differentiate between harmless and hazardous substances in wastewater, demonstrating the potential for cost-effective and efficient monitoring solutions. The integration of IoT technologies in water management is further explored in [14], which uses ARIMA and HOT-SAX techniques to detect anomalies in consumption patterns from smart meters. This approach aims to prevent water waste and optimize resource use. In [15], a novel algorithm for real-time anomaly detection leverages historical patterns and prediction intervals to reduce false alarms and improve performance. The study emphasizes balancing time and space complexity to enhance the algorithm’s efficiency. Additionally, ref. [1] introduces MCN-LSTM, a deep learning-based technology for real-time water quality monitoring, achieving high accuracy in anomaly detection. This advancement supports decision-making and environmental protection in automated monitoring systems. Finally, in [16], the authors present a prototype water quality monitoring system based on IoT technologies, incorporating a LoRa repeater and an anomaly detection algorithm. By extending LoRa communication coverage and improving long-range wireless communication distance, the system enhances monitoring reliability while addressing communication and detection challenges. In conclusion, these studies collectively underscore the importance of developing sophisticated, reliable, and efficient methods for anomaly detection in water quality monitoring. The integration of machine learning, IoT technologies, and advanced algorithms offers promising solutions to address the challenges of environmental monitoring and protection.

## 3. Anomaly Detection

In this section, the background of anomaly detection is initially established, outlining the different types, before being linked to the methodology used in this work.

An anomaly is a collection of occurrences that deviate significantly from the remainder of the dataset. They are often referred to as outliers, novelties, or noise. Measurement variability or certain experimental mistakes that are often removed from the dataset might result in an outlier [17].

An anomaly might result in incorrect conclusions and major issues with data analysis. Anomalies might enter the dataset for a number of causes. For example, hardware that is utilized to take measurements may briefly malfunction. Data transfer mistakes might happen from time to time. In addition, system behavior variations, instrument malfunctions, human influences, and dishonest activity can all result in anomalies.

### 3.1. Anomaly Detection Framework

**Figure 1:**
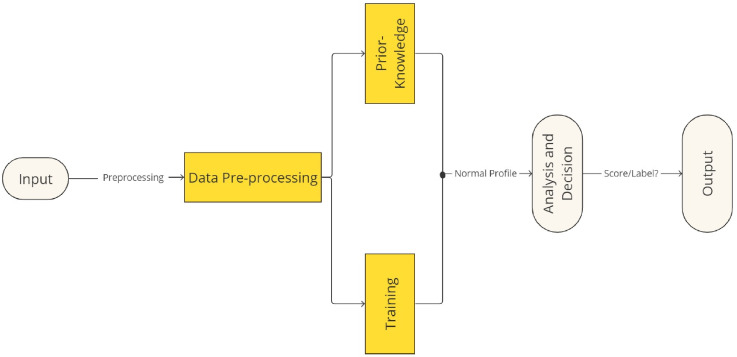
Framework of Anomaly detection.

A general anomaly detection framework [18] is depicted in Figure 1 and was developed from a generic intrusion detection framework. A dataset of data instances with data characteristics is the input used in an anomaly detection system. The data may need to be pre-processed before continuing, depending on the detection strategy. After that, data processing is used to create a normal profile by applying either a training technique or past knowledge. An established threshold can be used to identify a test instance as anomalous once a normal profile has been created.

Several indicators may be used to classify anomaly detection techniques. A common indicator used to categorize them is the model employed in detection techniques. In this context, parametric (or statistical) and non-parametric models are the two general types of models [19]. When the data distribution is well-known and unlikely to vary often, parametric approaches are appropriate in stable contexts. Non-parametric models can be applied in dynamic settings with new and uncertain statistical distributions. Faster detection is offered by the first category, while detection generality is provided by the second. A comparison of parametric and non-parametric systems is presented in Table 1.

**Figure 2:**
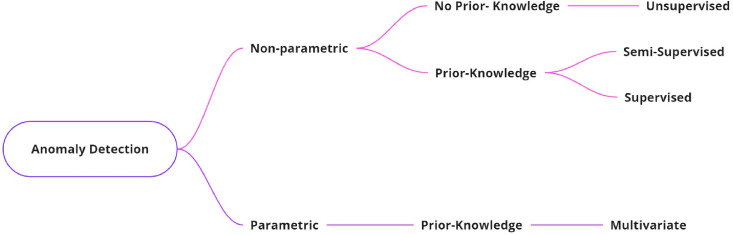
Taxonomy of anomaly detection.

Additionally, there are two general categories of anomaly detection schemes: those that need previous knowledge of the system data and those that do not [20]. Similar to the prior classification, the first category offers quicker detection, and the second category offers more general detection. Supervised or semi-supervised learning can be used by the classifier in the first category [21]. When a system’s typical behavior changes, the former requires a large training set and requires retraining, but the latter generalizes findings from a smaller dataset and can instantly adjust to system changes. The second category employs unsupervised learning and makes the assumption that aberrations are quite different from the norm. It does not require prior information. Figure 2 provides a summary of this section and compares the various training techniques in Table 1. This work concentrates on one type since it highlights the data that designers must have before using the algorithm, which is important for anomaly detection techniques as will be discussed later.

There are three general types of anomalies: point anomalies, contextual anomalies, and collective anomalies [18].

#### 3.1.1. Point Anomalies

A single data point that is isolated from other data points and is deemed unusual is called a point anomaly. Point anomalies are the most basic kind of anomaly and are often referred to as global anomalies. The majority of research focuses mostly on point anomaly detection.

**Figure 3:**
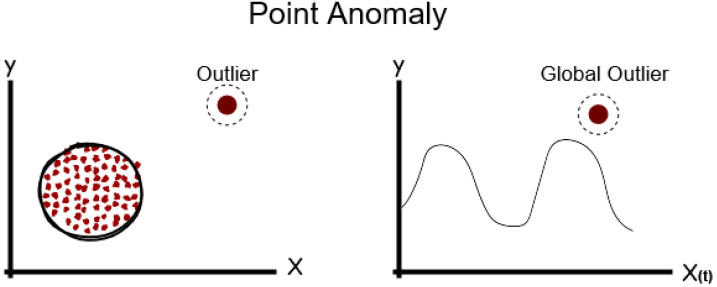
Point anomaly [22].

The most basic kind of anomalies are point anomalies, commonly referred to as global anomalies. Most research focuses on point anomaly detection. As shown as in Figure 3. Being distinct from other normal data points, the red point is a point anomaly as it is located beyond the borders of the normal data cluster, which are represented by the grey points.

#### 3.1.2. Contextual Anomalies

**Figure 4:**
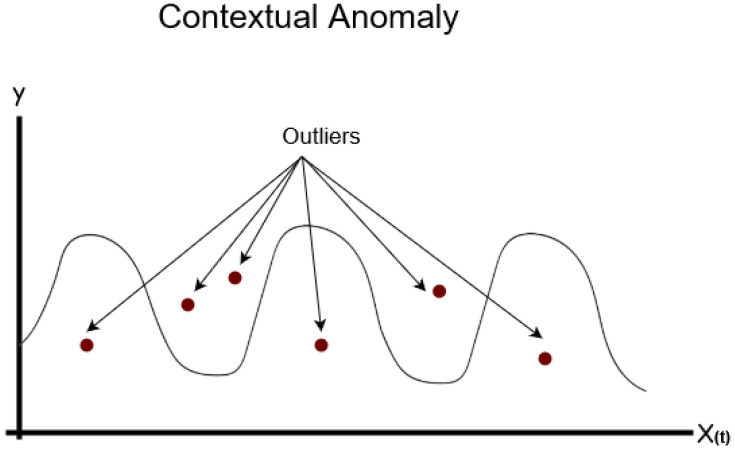
Contextual Anomaly [22].

Contextual anomalies are those instances that are deemed abnormal within a specific context. A point is examined in several settings in order to ascertain whether or not it is abnormal. For instance, the context is nearly always temporal in the case of time-series data, which are recordings of a certain amount across time. Contextual outliers, on the other hand, are data points that significantly deviate from other data within the same context. Anomalous observations in a time series are those that deviate considerably from the observed pattern and do not follow the pattern in the time-series data. Figure 4 shows an example for contextual anomalies.

#### 3.1.3. Collective Anomalies

An anomalous collection of data points is one that includes associated, linked, or sequential examples that dramatically deviate from the rest of the data.

**Figure 5:**
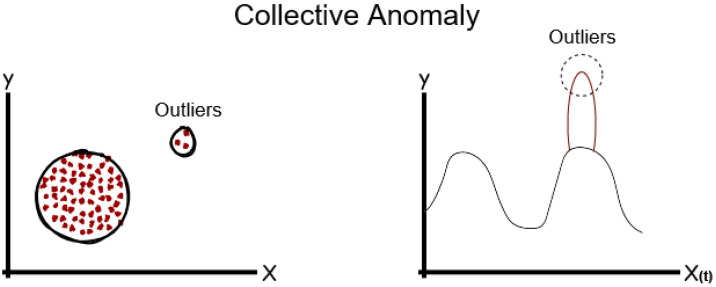
Collective Anomaly [22].

When a subset of data points in a data collection have values that collectively deviate significantly from the values of the entire dataset but the individual data points’ values are normal in both a contextual and global sense, this is referred to as a collective anomaly. In Figure 5 showns a group of points.

## 4. Sensing Parameters

**Figure 6:**
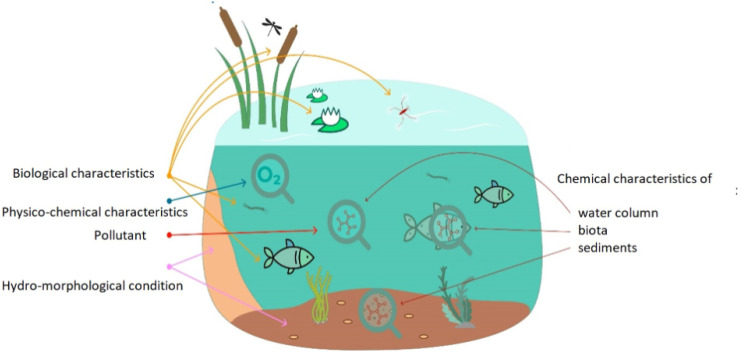
Water quality described by the physical, chemical, and biological characteristics [24].

Water quality is described by the physical, chemical, and biological characteristics of water (Figure 6). It is a measure of the condition of water relative to the requirements of one or more biotic species and or to any human need or purpose. The most common standards used to assess water quality relate to the health of ecosystems, safety of human contact, and drinking water. The physical quality of water affects the aesthetic quality of water in the context of appearance, color, taste, foam, smell, EC, turbidity, total dissolved solids, and temp. Besides that, the chemical quality of water is used to determine the concentration of the dissolved chemicals and is crucial to ascertain its health and condition. Finally, the microbiological quality of water describes the occurrence of invisible organisms in water, such as bacteria and viruses. The microbes are contaminants that cause devastating effects on the health of humans when the water containing these microbes is ingested. Measuring each parameter is in most of the cases unnecessary. One way to track the status and changes of these dynamic systems is through indicators. An indicator represents the state or trend of specific environmental conditions over a given area and a specified period. For example, for the National Aquatic Resource Surveys (NARS), biological, chemical, and physical indicators were chosen to assess biological integrity, trophic state, recreational suitability, and key stressors impacting quality. Although there are more indicators and stressors, these are among the most representative nationally. Monitoring the temp, EC, turbidity, and DO level in conjunction with the pH deliver enough information on whether sanitation is required or, for example, if public access should be granted [23].

**Figure 7:**
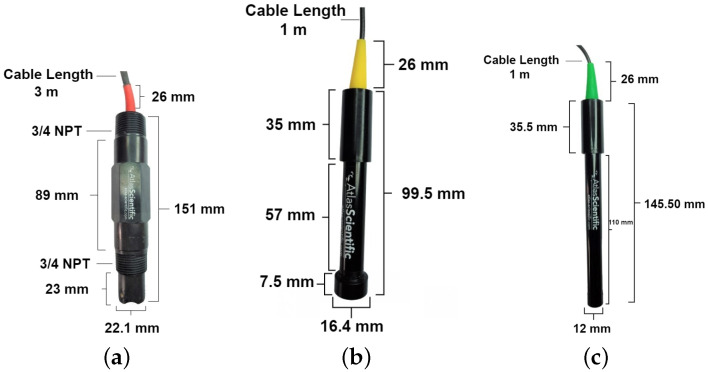
pH and temperature sensor (**a**). Dissolved oxygen sensor (**b**). Electrical conductivity sensor (**c**).

In this section, we provide an overview of the sensor used in this research (Figure 7). We employed four sensors sourced from Atlas-Scientific, Long Island, NY, USA, a company specializing in water monitoring and environmental robotics, offering laboratory-grade accuracy. These sensors will detect the following chemical indicators:

- pH
- Temp
- EC
- DO

The main sources of chemicals that reduce pH are acid rain and acid mine drainage. On the other hand, significant concentrations of organic substances or the presence of flora that produces acid can also cause this to occur naturally. Most living things are found in the pH range of 6.5 to 8.5. Numerous species, such as neutrophile bacteria, are susceptible to pH disturbances. Air and aquatic plants provide oxygen to water resources. Compared with motionless water in a pond or lake, running water dissolves more oxygen. Microorganisms may break down surplus organic materials, such massive algal blooms, to produce low oxygen levels (hypoxia), or no oxygen at all (anoxia). During this decomposition process, oxygen in the water is consumed. As DO clearly shows whether an aquatic resource can sustain aquatic life, it is regarded as a crucial indicator of water quality. Turbid waters absorb and scatter light, whereas clear waterways have minimal amounts of suspended soil particles or algae. Water purity affects the diversity and productivity of aquatic life significantly, as it is directly tied to light penetration. For instance, more sunshine can reach submerged aquatic plants in cleaner water. Thus, the vegetation creates food for fish, animals, and ducks, as well as habitats for fish and shellfish. It also produces oxygen. Furthermore, people often favor clean waterways for recreational and aesthetic reasons. Natural variations in wind patterns, sunshine levels, tides, and storm activity can all affect the purity of the water. It is impacted by a number of physical, chemical, and biological elements related to the natural geology and human usage of the surrounding watershed. Certain waterways have turbidity by nature. However, water that is very nutrient-rich (from fertilizers, for example) and sediment-rich (from building materials, for example) tends to be murky and less translucent. Conductivity rises with salinity because dissolved salts and other inorganic substances conduct electricity. Oil and other organic molecules, on the other hand, have a lower conductivity. Every body of water has a generally consistent range of conductivity, which can serve as a reference point for doing routine conductivity tests. Considerable changes in conductivity may be a sign that the aquatic resource has been contaminated by another source. Table 2 lists the used parameters and their standards.

## 5. Methodology

This section provides an overview of the methodology used to handle the data according to the approach utilized for outlier detection, based on four sensors highlighted in Section 4. These sensors are part of a real-time monitoring system designed for smart sensing of water bodies lakes, rivers, and ponds in Brussels. The collected data consist of environmental parameters collected and then sent to cloud infrastructure.

Our methodology starts with the data collecting process that involves using LoRa technology for wireless communication, where each sensor transmitted their observations to a central gateway. Subsequently, the collected data are transmitted to the server for in-depth analysis via the LoRaWAN protocol.

After receiving the data on the server, our analysis pipeline begins with data storage, preceding the division of the dataset into an 80% portion for training and a 20% portion for testing. Prior to the split operation, the data labeling process are performed before applying the machine learning models. Subsequently, we evaluated the used models relying on $F_{1}$-score.

**Figure 8:**
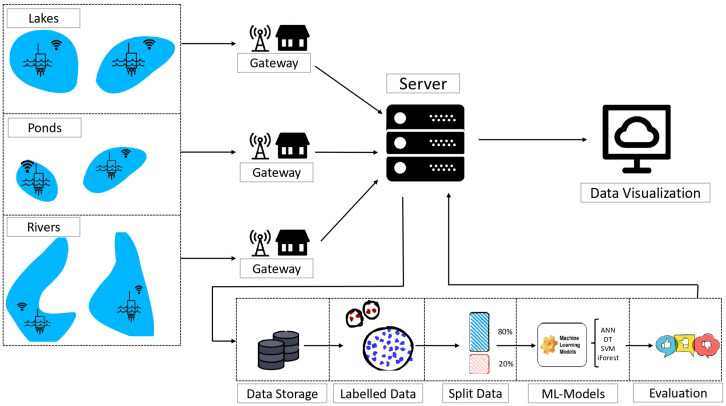
Steps Involved in the data collection, pre-processing, and data visualization process.

Finally, the phase of data visualization involved to clearly represent the collected data, ensuring an accurate interpretation and actionable insights. Figure 8 serves as an illustration of this process.

### 5.1. Deployment

**Figure 9:**
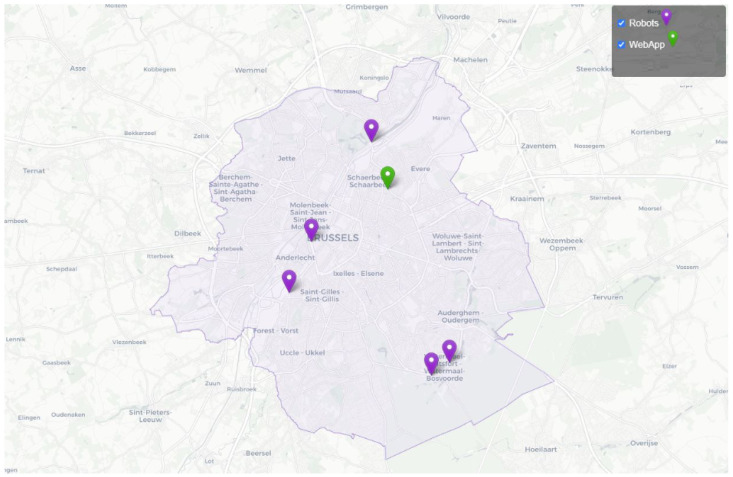
Map of Brussels highlighting areas where samples were taken.

**Figure 10:**
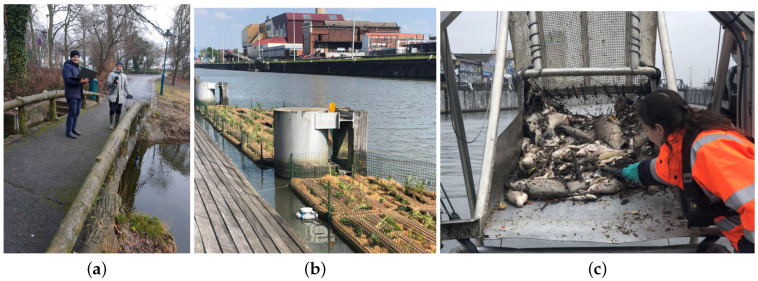
Collecting samples from the leybeek ettang (**a**). Brussels canal (**b**). Death fish found on the canal (**c**).

In Brussels, various types of water bodies exist, including lakes, rivers, and ponds. Samples have been taken from five locations to test the sensors in different environments, as shown in Figure 9. These locations include the Brussels Royal Yacht Club (BRYC) on the Brussels canal (Figure 10b), the Brussels Sewer Museum, Wiels Swamps, Leybeek Ponds (Figure 10a), and the pond at Tournay-Solvay Park. A large number of dead fish were found floating on the surface of the Brussels canal (Figure 10c). During this period, significant increases in temperatures and sudden drops in DO levels were observed. Temp is a physical parameter that affects the equilibrium of the aquatic environment, influencing water density and altering water flow speed. Moreover, rising temp reduces the amount of DO in water, potentially leading to anoxia. Elevated water temp can also stimulate bacterial growth, which may contribute to the spread of diseases within the aquatic ecosystem [27,28]. Additionally, wastewater was discharged into the canal on that day, exacerbating the issue.

### 5.2. Datasets Selection

To enable the recognition of anomalies, classifiers used for sensors require training. For this purpose, datasets containing labeled water sensor information are crucial. The dataset utilized in this research was gathered from various locations via robots manufactured in our laboratory. This dataset consists of 2971 rows of data, and 4 columns, representing data from 14 robots, each transmitting the data to the cloud every 10 min; furthermore, the datasets are heterogeneous enough to be representative for the water sensors.

### 5.3. Fine-Tuning and Classifiers

There are several classical machine learning (ML) techniques that can perform well in the context of anomaly detection. For this study, supervised ML techniques, which use correctly labelled data during the classifiers’ training, are used. They are known to deliver a good performance when classifying the data of sensors. These are the selected supervised ML techniques:

- ANN: It is a set of interconnected nodes designed to imitate the functioning of the human brain. Each node has a weighted connection to several other nodes in neighboring layers. Individual nodes take the input received from the connected nodes and use the weights together with a simple function to compute output values. Neural networks can be constructed for supervised or unsupervised learning. The user specifies the number of hidden layers as well as the number of nodes within a specific hidden layer. Depending on the application, the output layer of the neural network may contain one or several nodes. The multilayer perceptions (MLP) neural networks have succeeded in various applications and produced more accurate results than other computational learning models. They can approximate any continuous function to a random accuracy as long as they contain enough hidden units. Such models can form any classification decision boundary in feature space and thus act as a non-linear discriminate function [29]. ANN is known to be a good technique for the classification of complex datasets. In this study, we used ANN with two hidden layers and an output layer with a single neuron for binary classification, where the ReLu activation function is used in the hidden layer and a sigmoid activation function is used in the output layer. To train the model, we used the Adam optimizer, with a learning rate equal to 0.0001 and regularization parameter equal to 0.1.
- SVM: SVM is a supervised machine learning algorithm introduced by [30], which can be used for classification and regression problems. The SVM technique separates the data belonging to different classes by fitting a hyperplane between them, which maximizes the separation. The data are mapped into a higher dimensional feature space where a hyperplane can easily separate it. Furthermore, a kernel function approximates the dot products between the mapped vectors in the feature space to find the hyperplane. In addition, using the binary classification approach, SVM uses one-class quarter sphere to reduce the effort of computational complexity and locally identify outliers generated by the sensors. Consequently, the sensor data outside the quarter sphere are considered as the outlier [31]. We tested different variants using SVM by changing the parameters kernel, regularization parameter, and gamma. After parameter tunning, kernel = ‘rbf’, regularization parameter = 0.1, and gamma = 0.001 achieved one of the best $F_{1}$-score.
- DT: The DT algorithm is considered a powerful technique for detecting and manipulating outliers within datasets. It is a type of supervised machine learning algorithm that can be used for both classification and regression. When a DT is trained on datasets, it learns to recursively split the data based on feature values that minimize some measure of impurity [32]. During this process, the DT may isolate certain observations into internal nodes (leaf nodes) that are distinct from the majority of data points. These isolated instances could potentially be outliers. The DT algorithm can be effectively used for outliers detection because of its capability to partition the feature space and isolate instances that deviate significantly from the majority of data points [33]. In this study, we used DT with its default parameters, because after the fine tuning of its parameters, the model provided approximately the same values. In this context, we decided to go with the default parameters: Nestmators = 50, MinSampleSplit = 2, MinSampleLeaf = 1, Maxfeatures = auto.
- iForest: The iForest algorithm relies on creating a set of binary decision trees. Unlike the decision trees, the iForest trees randomly selected features and split points to create partitions. This process of randomly finding the trees can sometimes be powerful as outliers are often located in sparsely populated regions of the feature space. More specifically, the isolation forest algorithm gives an isolation score to each data point based on its average path length across all the trees in the forest. Data points with shorter average path length are considered outliers, as they demand fewer splits to isolate [34]. For the iForest, we used contamination with a value of 0.1 and N-estimators with 50 as the value.

### 5.4. Metrics

To conduct a comprehensive assessment of the various classifiers, a variety of metrics, including accuracy, are utilized to ensure their thorough evaluation. On the one hand, fair comparison of the classifiers is achieved using accuracy measurements like the $F_{1}$-score [35,36,37].

#### Accuracy

The evaluation of the accuracy utilized the $F_{1}$-score, a metric well-suited for multi-classifiers and imbalanced data. The accuracy and recall metrics, which are based on true positives, false positives, and false negatives, are harmonic means that provide the $F_{1}$-score. Precision (Equation (1)) evaluates a classifier’s ability to correctly identify instances belonging to each class and recall (Equation (2)) is the classifier’s capacity to locate every accurate instance in each class. They are expressed as follows:

(1) $$Precision\;=\;\frac{t_{p}}{t_{p}+f_{p}}$$

(2) $$Recall\;=\;\frac{t_{p}}{t_{p}+t_{n}}$$

where $t_{p}$ and $t_{n}$ are the number of true positives and true negatives, respectively, while $f_{p}$ is the number of false positives. Both parameters are used to obtain the $F_{1}$ score as follows:

(3) $$F_{1}\;score\;=\;\frac{2\times Precision\times Recall}{Precision+Recall}$$

The value of the $F_{1}$-score is normalized between 0 and 1, with a score of 1 indicating a perfect balance, as precision and recall are inversely related [38]. Moreover, the $F_{1}$-score takes into account imbalanced class distribution, such as that which occurs with the evaluated classifiers in Section 7.

The micro average ($F_{1}$-micro), macro average ($F_{1}$-macro), and weighted average ($F_{1}$-weighted) are three techniques for averaging the $F_{1}$-score. The performance is calculated by the $F_{1}$-micro using the individual true positives, false positives, and false negatives. The $F_{1}$-micro can be a deceptive statistic in situations where the class distribution is unbalanced, such as in our dataset, because it is not sensitive to the correctness of individual classes. When a classifier has a high micro average, it indicates a good overall performance. The performance of individual classes is used to calculate the $F_{1}$-macro, which is based on bigger groupings. As a result, it works better when the data show an unequal distribution of classes. When the $F_{1}$-macro is high, the classifier works well for every single class. The $F_{1}$-weighted is computed using the individual class performance, the same as the $F_{1}$-macro, but it modifies the outcome to take label imbalance into consideration [39]. In this evaluation of classifiers, we utilize the $F_{1}$ score.

## 6. Evaluation and Platforms

In this section, we provide details on the pre-processing of sensor data related to water technology, the training procedure, and the platforms and libraries utilized.

### 6.1. Dataset Pre-Processing

The dataset mentioned earlier in Section 5.2 consists of 2971 rows and 4 columns. It is divided into two parts: 80% for training and 20% for testing. There is no dataset augmentation applied. An additional column representing labels is appended to the dataset, resulting in a total of 5 columns. The labels are generated based on Table 2.

### 6.2. Platforms and Experimental Setup

In this section, we provide an overview of the various tools and materials used in our research. The Python 3.10 package includes the Scikit-learn 1.4 library, which is instrumental in implementing our models. Additionally, we utilize the TensorFlow framework, which plays a crucial role in developing, training, and deploying our models. Moreover, we employ the ChirpStack (v4) platform as an open-source LoRaWAN server, facilitating communication between LoRaWAN devices and applications. The data from robots are stored in InfluxDB, a time-series database suitable for IoT applications. Additionally, InfluxDB enables us to monitor and analyze data. However, for this research, we use Grafana. This powerful visualization and analytics platform provides us with numerous options to create intuitive and interactive dashboards. We utilize version 10.1.17 of Grafana for visualization purposes. The laptop used in the experiments features an Intel(R) Core(TM) i7-9850H CPU (Intel, Santa Clara, CA, USA) running Windows 10.

## 7. Results and Discussion

In this section, a comparison will be conducted among four sensors—pH, temperature, EC, and DO—utilizing four different models—SVM, iForest, DT, and ANN.

Considering the results presented in Table 3, it is clearly seen that the SVM model achieved excellent results in the outlier detection context relying on pH measures. It exhibited high precision, recall, and $F_{1}$-score on the training and test sets, with a notably high $F_{1}$-score (0.9838) on the test set. Conversely, the iForest showed lower precision, recall, and $F_{1}$-score across training and test sets, presenting limitations in effectively identifying outliers. The decision tree model performed well, boasting high precision and reasonably good recall and $F_{1}$-score. While ANN displayed good precision and recall values, its $F_{1}$-score was comparatively lower than SVM and DT, particularly on the test set. As a result, using a simpler model such as DT or SVM might yield superior results, unless additional optimization or fine-tuning enhances the performance of the ANN.

As depicted in Table 4, SVM showcased strong capabilities in detecting outliers based on temperature measures, maintaining high precision, recall, and $F_{1}$-scores on the training and test sets. On the other hand, iForest displayed a moderate performance, with lower precision, recall, and $F_{1}$-scores compared with SVM. The decision tree exhibited an acceptable capability, though slightly lower than SVM. Although slightly lower than SVM, ANN showed a competitive performance, with solid accuracy, recall, and $F_{1}$-scores.

By analyzing the results of Table 5, SVM demonstrated a strong performance in outlier detection based on DO measures, maintaining consistently high precision, recall, and $F_{1}$-scores on both the training and test sets. iForest displayed lower precision, recall, and $F_{1}$-scores compared with SVM. DT exhibited a decent performance, slightly lower than SVM but still effective. ANN demonstrated a strong and competitive performance, similar to SVM. SVM and ANN emerged as top performers in outlier detection based on DO measures, demonstrating consistently high precision, recall, and $F_{1}$-scores on both the training and test sets.

Concerning the electrical conductivity in Table 6, SVM demonstrated a robust performance in outlier detection, showcasing high precision, recall, and $F_{1}$-scores on both the training and test sets. Isolation Forest displayed lower precision, recall, and $F_{1}$-scores than SVM. In the same context, the decision tree also lower values than SVM. ANN is closely aligned with SVM. Therefore, SVM and ANN can be considered the best solutions in the context of outlier detection based on conductivity measures, demonstrating consistently high precision, recall, and $F_{1}$-scores on both the training and test sets.

Overall, the analysis of different measures of pH, temp, EC, and DO revealed that SVM consistently performed well in outlier detection across the different measures, showcasing strong precision, recall, and $F_{1}$-scores on both the training and test sets. The ensemble methods of decision tree and ANN also exhibited a competitive performance, indicating potential alternatives depending on specific requirements.

In this study, we evaluated four machine learning models—SVM, iForest, DT, and ANN—to assess their effectiveness in predicting critical water quality parameters—pH, temperature, DO, and EC. Our evaluation focused on interpreting the implications of the models’ performance metrics, particularly $F_{1}$-scores, across both the training and test datasets. Across all of the evaluated parameters, SVM consistently emerged as the top-performing model in terms of $F_{1}$-score. This metric combines precision and recall, offering a balanced measure of a model’s predictive capability. SVM’s robust $F_{1}$-scores on both training and test datasets indicate its reliability in accurately predicting water quality metrics. The superior performance of SVM can be attributed to several factors inherent to its algorithmic design. SVM effectively constructs hyperplanes or decision boundaries that maximize the margin between different classes, leading to strong generalization capabilities. This attribute is particularly advantageous in complex datasets where a clear separation between classes is critical.

iForest, DT, and ANN also demonstrated a competitive performance across specific parameters. However, their overall $F_{1}$-scores were consistently lower than SVM. Isolation forest, despite its anomaly detection strength, struggled to capture the complex patterns present in water quality data, resulting in comparatively lower precision and recall. Decision Tree and ANN, while showing promise in certain metrics, did not consistently match SVM’s robustness across all parameters.

The choice of SVM as the preferred model for water quality prediction bears significant practical implications. Its ability to maintain high accuracy and reliability across diverse datasets suggests its suitability for deployment in real-world applications such as environmental monitoring and management. Reliable predictions of pH, temperature, dissolved oxygen, and EC are crucial for decision-making processes aimed at safeguarding aquatic ecosystems and public health.

Despite SVM’s strong performance, there are avenues for further exploration. Future research could delve into optimizing SVM parameters or exploring ensemble methods to enhance its predictive accuracy further. Additionally, investigating the scalability of SVM to larger datasets or its adaptability to changing environmental conditions would be beneficial for extending its applicability.

In conclusion, our comparative analysis underscores SVM’s effectiveness as a robust predictive model for water quality parameters. Its consistent performance across multiple metrics and datasets positions SVM as a valuable tool in advancing environmental informatics, offering reliable insights into water quality dynamics crucial for sustainable resource management and ecosystem preservation. Future research efforts should continue to explore and refine SVM’s capabilities to address the evolving challenges in water quality assessment and monitoring.

## 8. Conclusions

Poor water quality is a severe problem threatening human health, ecosystems, plants, and animals. Therefore, monitoring water quality has become a primary concern in water resources and environmental systems. Traditionally, to monitor water quality, manual laboratory-based testing can be costly, time-consuming, and lack real-time feedback. For this reason, the intersection of technology and environmental sustainability has turned into a necessity in order to ensure the efficient monitoring and controlling of water. In this context, using smart water sensing technology has become a pivotal milestone in managing water, relying on the data collected by different sensors and deployed in various aquatic environments. However, the collected data are confounded by anomalies caused by technical issues, which can lead to fault detection and diagnosis. In light of this challenge, this work specifically focused on the detection of outliers to ensure the efficient management of water using machine learning techniques. As a result, the monitoring of four key sensors, namely, PH, DO, EC, and Temp, are each evaluated through the lens of four essential classical machine learning algorithms: support vector machine, artificial neural network, decision tree, and isolated forest-based outlier detection.

The dataset utilized in this study originates from a real-time smart water sensing monitoring system deployed across different environments, including lakes, rivers, and ponds in Brussels. The experimental study has shown the superior efficiency of SVM, which demonstrates an exceptional $F_{1}$-score rate across multiple sensors. In addition, we highlight the noteworthy performance of ANN, suggesting its potential capability as a main alternative to address the problem of outlier detection.

## Figures and Tables

**Table 1:** Comparison of anomaly detection training methods.

	Supervided	Semi-Supervised	Unupervised
Prior-Knowledge?	Yes	Yes	No
Environment?	Static	Dynamic	Dynamic
Detection speed?	fast	Fast/Moderate	Moderate/slow
Detection generality?	No	Yes	Yes

**Table 2:** Water quality standards provide by World Health Organization.

Parameters	Units	WHO Standards [25]	Analytical Methods [26]
pH	-	6.5–8.5	pH meter
EC	μs/cm	400	Electrometric
Temp	°C	12–25	Digital thermometer
DO	mg/L	4–6	Winkler method

**Table 3:** $F_{1}$ scores for pH are measured for the four models. The solution with the highest $F_{1}$ score is indicated.

Models		Precision	Recall	$F_{1}$-Score
SVM	Train	0.9599	1	0.9796
	Test	0.9681	1	0.9838
iForest	Train	0.775487	0.82154	0.7978
	Test	0.754875	0.800014	0.7767
DT	Train	0.9599	0.97002	0.9649
	Test	0.9681	0.96004	0.9640
ANN	Train	0.9	0.95	0.9243
	Test	0.8925	0.9422	0.9167

**Table 4:** $F_{1}$ scores for temperature is measured for the four models. The solution with the highest $F_{1}$ score is indicated.

Models		Precision	Recall	$F_{1}$-Score
SVM	Train	0.97145	0.984421	0.9778
	Test	0.95998	0.97999	0.9698
iForest	Train	0.7823	0.825574	0.8033
	Test	0.76545	0.8021254	0.7833
DT	Train	0.88	0.9	0.8898
	Test	0.8732	0.8854	0.8792
ANN	Train	0.94	0.96	0.9498
	Test	0.9354	0.9588	0.9469

**Table 5:** $F_{1}$ scores for dissolved oxygen is measured for the four models. The solution with the highest $F_{1}$ score is indicated.

Models		Precision	Recall	$F_{1}$-Score
SVM	Train	0.9832	0.9745	0.9788
	Test	0.9720	0.9610	0.9666
iForest	Train	0.8054	0.7899	0.7976
	Test	0.7921	0.7885	0.7903
DT	Train	0.9	0.92	0.9098
	Test	0.899	0.9125	0.9076
ANN	Train	0.9877	0.9751	0.9813
	Test	0.9835	0.9742	0.9788

**Table 6:** $F_{1}$ scores for EC is measured for the four models. The solution with the highest $F_{1}$ score is indicated.

Models		Precision	Recall	$F_{1}$-Score
SVM	Train	0.98400	0.97897	0.9814
	Test	0.9752	0.9695	0.9723
iForest	Train	0.8288	0.7756	0.8013
	Test	0.72400	0.7766	0.7494
DT	Train	0.8920	0.8854	0.8871
	Test	0.8912	0.8766	0.8839
ANN	Train	0.9900	0.9845	0.9872
	Test	0.9802	0.9820	0.9811

## Data Availability

The data are available upon request.
